# Gut Microbiota-Derived Tryptophan Metabolite Indole-3-aldehyde Ameliorates Aortic Dissection

**DOI:** 10.3390/nu15194150

**Published:** 2023-09-26

**Authors:** Sui-Shane Huang, Rongle Liu, Shufu Chang, Xiao Li, Xinyu Weng, Junbo Ge

**Affiliations:** 1 Department of Cardiology, Zhongshan Hospital, Fudan University, Shanghai Institute of Cardiovascular Diseases, Shanghai 200032, China; 16111210100@fudan.edu.cn (S.-S.H.); liu.rongle@zs-hospital.sh.cn (R.L.); chang.shufu@zs-hospital.sh.cn (S.C.); li.xiao1@zs-hospital.sh.cn (X.L.); 2 Key Laboratory of Viral Heart Diseases, National Health Commission, Shanghai 200032, China; 3 Key Laboratory of Viral Heart Diseases, Chinese Academy of Medical Sciences, Shanghai 200032, China; 4 National Clinical Research Center for Interventional Medicine, Shanghai 200032, China

**Keywords:** aortic dissection, indole-3-aldehyde, smooth muscle cell, inflammation, extracellular matrix degradation

## Abstract

Tryptophan, an essential dietary amino acid, is metabolized into various metabolites within both gut microbiota and tissue cells. These metabolites have demonstrated potential associations with panvascular diseases. However, the specific relationship between tryptophan metabolism, particularly Indole-3-aldehyde (3-IAId), and the occurrence of aortic dissection (AD) remains unclear. 3-IAId showed an inverse association with advanced atherosclerosis, a risk factor for AD. In this study, we employed a well-established β-aminopropionitrile monofumarate (BAPN)-induced AD murine model to investigate the impact of 3-IAId treatment on the progression of AD. Our results reveal compelling evidence that the administration of 3-IAId significantly mitigated aortic dissection and rupture rates (BAPN + 3-IAId vs. BAPN, 45% vs. 90%) and led to a notable reduction in mortality rates (BAPN + 3-IAId vs. BAPN, 20% vs. 55%). Furthermore, our study elucidates that 3-IAId exerts its beneficial effects by inhibiting the phenotype transition of vascular smooth muscle cells (VSMCs) from a contractile to a synthetic state. It also mitigates extracellular matrix degradation, attenuates macrophage infiltration, and suppresses the expression of inflammatory cytokines, collectively contributing to the attenuation of AD development. Our findings underscore the potential of 3-IAId as a promising intervention strategy for the prevention of thoracic aortic dissection, thus providing valuable insights into the realm of vascular disease management.

## 1. Introduction

Aortic dissection (AD) is a critical medical condition characterized by a tear in the intimal layer of the aorta or bleeding within the aortic wall, resulting in the separation (dissection) of the layers of the aortic wall [1]. This disease exhibits rapid progression and is associated with a high mortality rate [2]. Within 24 h of AD onset, the fatality rate reaches 50.0%, increasing to 68.2% after 48 h [3]. There is an urgent need to develop effective treatment strategies, as no clinically proven drugs are available to prevent or delay the progression of AD.

The pathological characteristics of abdominal aortic aneurysm (AAA) primarily encompass an inflammatory response, apoptosis of vascular smooth muscle cells (VSMC), the impairment of vascular endothelial cells (VECs), the induction of oxidative stress, and degradation of the extracellular matrix (ECM) [4]. In the aortic media, an infiltration of various leukocytes and inflammatory mediators, including interleukin (IL)-1, IL-17, and transforming growth factor (TGF)-β, is observed. This infiltration leads to the transformation of smooth muscle cells, the generation of reactive oxygen species (ROS), and the activation of matrix metalloproteinases (MMPs). Consequently, these processes culminate in the fragmentation of the extracellular matrix (ECM) [5]. The shift from the contractile to the synthetic VSMC phenotype, triggered by PDGF, initiates upon PDGF binding to surface receptors. This binding activates diverse intracellular signaling pathways that ultimately regulate gene expression and cellular functions. These pathways have been shown to result in a decrease in contractile protein levels and an increase in the expression of synthetic proteins, such as osteopontin [6,7] and vimentin [8,9], therefore leading to the disruption of the aortic wall.

The gut microbiome, comprising trillions of commensal organisms, functions as a metabolically active endocrine-like organ and plays a pivotal role in host physiology. It actively contributes to macronutrient digestion, vitamin synthesis, and the generation of biologically active metabolites [10]. Indole, which is produced by a variety of symbiotic bacteria encompassing both Gram-positive and Gram-negative strains, including *Escherichia coli*, *Prevotella*, and *Bacteroides* [11], has been implicated in abnormal tryptophan metabolism. This abnormality leads to an elevation in the levels of 3-hydroxy-o-aminobenzoic acid via the transcription factor NF-κB, subsequently upregulating MMP2 and contributing to the occurrence of AAA [12]. Tryptophan metabolites exhibit potent inflammatory and anti-inflammatory effects, thereby potentially influencing the development of atherosclerosis and aneurysms. Consequently, the tryptophan metabolic pathway emerges as a promising target for AAA treatment [13]. Microbiota-derived tryptophan metabolites also directly affect the vascular endothelium, impacting the development of vascular inflammatory phenotypes [14]. Indoxyl sulfate promotes vascular inflammation [15], while indole-3-propionic acid and Indole-3-aldehyde (3-IAId) have protective roles [16,17]. Furthermore, there is increasing evidence for the contributory role of microbiota-derived indole-derivatives in blood pressure regulation and hypertension [18,19]. Additionally, there are indications for a function of the kynurenine pathway in atherosclerotic lesion development [20].

Despite extensive investigation of tryptophan metabolites in relation to cardiometabolic diseases, the role of the tryptophan metabolite 3-IAId in AD remains significantly understudied. In our study, we aim to investigate the role of 3-IAId in the progression of aortic dissection.

## 2. Materials and Methods

### 2.1. Animals

Male C57BL/6N mice at the age of three weeks were procured from the Shanghai JieSiJie Laboratory Animal Center (Shanghai, China). These mice were accommodated in individually ventilated cages, maintaining pathogen-free conditions, and provided unrestricted access to both food and water. Monitoring encompassed assessments of body weight, behavioral patterns, as well as food and water consumption to ensure their overall well-being. Ethical approval for all animal procedures was obtained from the Committee on the Ethics of Animal Experiments at Zhongshan Hospital, Fudan University.

To induce aortic dissection (AD), the three-week-old male C57BL/6 mice were subjected to a 28-day regimen of β-aminopropionitrile monofumarate (BAPN, 0.5%; Sigma-Aldrich, St. Louis, MO, USA) in their drinking water [21]. Simultaneously, Indole-3-aldehyde (3-IAId, 487898, Sigma, Burlington, MA, USA), dissolved in Dimethyl sulfoxide (DMSO, 67-68-5, Sigma, Burlington, MA, USA), was administered via oral gavage at a dosage of 150 mg/kg/day. This 3-IAId solution was blended with DMSO (20%), polyethylene glycol 400 (PEG 400, 40%), and citric acid (2%) [22]. The mice were classified into four distinct groups: Control (*n* = 6), 3-IAId (*n* = 6), BAPN (*n* = 20), and BAPN + 3-IAId (*n* = 20). Following the 28-day experimental period, the mice were humanely euthanized, and their aortas were meticulously collected. Notably, all animals used in these experiments were male due to the reduced variability in sex hormones and the higher incidence of TAD in this gender.

### 2.2. Tissue Fixation and Paraffin Embedding

Fix the harvested tissues overnight at 4 °C in 4% paraformaldehyde (PFA) prepared in PBS, ensuring that the fixative volume is 10 times the tissue volume for optimal fixation. Following fixation, gently wash the tissues with PBS three times. Subsequently, store the tissues in 70% ethanol until they are ready for embedding. For dehydration, progress through the following ethanol concentrations with two changes for each step, each lasting one hour: 70% ethanol, 80% ethanol, 95% ethanol, and finally, 100% ethanol. Continue the process with three changes of xylene, each for one hour, and finish by immersing the tissues in paraffin wax (56–58 °C) for two changes, each for 1.5 h. Once the tissues are properly dehydrated and cleared, transfer them to embedding cassettes and proceed to embed them in paraffin blocks.

### 2.3. Hematoxylin and Eosin (HE) Staining, Elastic Van Gieson (EVG) Staining, and Immunofluorescence Staining (IF)

The mouse aortic tissues were cut into 5 µm thick serial sections, and the paraffin sections were stained after deparaffinization. Hematoxylin and eosin staining (G1120, Solarbio, Beijing, China) and elastic Van Gieson staining (G1593, Solarbio, Beijing, China) were performed on the aortic sections following the manufacturer’s instructions. Images of the stained sections were acquired using a bright field camera on a fluorescence microscope (Leica, Wetzlar, Germany). For the IF staining, the paraffin sections of the tissue were initially subjected to dewaxing, followed by microwave-assisted antigen retrieval. Following several rinses with PBS, the sections were enveloped with a solution comprising 5% bovine serum diluted in 1 × PBS. Subsequently, the paraffin sections were subjected to an overnight incubation at 4 °C with specific primary antibodies, including Mac2 (R&D systems, AF1197, 1:100, Minneapolis, MN, USA), Fibronectin (Abcam, ab2413, 1:100, Boston, MA, USA), and Collagen1A1 (Novus NBP1-30054, 1:50, Centennial, CO, USA). After additional washing with PBS, the paraffin sections were exposed to fluorescent secondary antibodies, specifically Alexa Fluor 488 rabbit Anti-goat IgG (Invitrogen, A11078, Carlsbad, CA, USA, 1:200), Alexa Fluor 568 donkey Anti-rabbit IgG (Invitrogen, A10042, Carlsbad, CA, USA, 1:200), and Alexa Fluor 488 donkey anti-rabbit IgG (Invitrogen, A32790, 1:200), for a duration of 1–2 h at room temperature. To preserve and seal the paraffin sections, an antifade mounting medium containing 4′,6-diamidino-2-phenylindole (DAPI, Abcam, ab104139) was employed. Immunofluorescence signals were subsequently visualized utilizing a confocal microscope (Zeiss, Jena, Germany).

### 2.4. Blood Pressure Measurement

After 4 weeks of BAPN modeling, the mice underwent noninvasive tail-cuff blood pressure measurements using the Softron bp-2010 mouse blood pressure monitor as previously described [23]. Following a 10 min adjustment period in a quiet and warm environment, the mice were placed into the mouse net, the heat preservation tube, and the ratbag. The pressure receptor was positioned at the tail root of the mouse with the marker in alignment with the tip of the mouse tail to measure SBP and DBP. Blood pressure values were recorded and averaged.

### 2.5. Gelatin Zymography Assay

Following the instructions, the Zymography Kit from COSMOBIO (Cat. No. AK47, Carlsbad, CA, USA) was utilized to detect ProMMP-2, MMP-2, ProMMP9, and MMP-9 in the aorta. Fresh aorta homogenates were gently collected and mixed in equal volume with non-reducing 2× loading buffer. Equivalent protein amounts were separated using a 10% SDS-PAGE gel, wherein gelatin solution (1%, 0.5 mL) was incorporated into the separation gel (10%, 5 mL). After electrophoresis, SDS removal from the separation gel involved three 25 min washes with a buffer containing 2.5% Triton X-100. Subsequently, the gel was incubated in an appropriate developing buffer (pH 7.6, 50 mM Tris-HCl, 10 mM CaCl_2_, and 5 mM NaCl) for 42 h at 37 °C. Coomassie Brilliant Blue was used for staining over a period of 2 h, followed by three washes with a de-staining solution. The gelatinolytic activity, indicative of MMP-2 and MMP-9 activity, appeared as white bands against a blue background.

### 2.6. Cell Culture

Human vascular smooth muscle cells (hVSMCs), sourced from the Cell Bank of the Chinese Academy of Sciences in Shanghai, China, were cultured in 6-well plates using a complete SMCM medium (ScienCell, Carlsbad, CA, USA), and maintained at 37 °C with 5% CO_2_ in a humidified incubator. The growth medium was supplemented with smooth muscle cell growth factor (SMCGS, ScienCell 1152), 10% fetal bovine serum (FBS, ScienCell 0010), and 1% penicillin/streptomycin (P/S, ScienCell 0503). Upon reaching confluence (80–90% confluency), the hVSMCs were subjected to treatment with 3-IAId (0.5 mM [17]; 24 h; Sigma–Aldrich, Burlington, MA, USA) and PDGF-BB (20 ng/mL [24]; 24 h; Sigma–Aldrich).

### 2.7. Western Blot

The frozen aortic tissues underwent homogenization through the utilization of an automated bead homogenizer. Total proteins were subsequently extracted from the aortic tissues by employing a RIPA buffer that contained a protease and phosphatase inhibitor cocktail (Cell Signaling Technology, 9806, Danvers, MA, USA). Following this, all of the protein samples for each experimental group were subjected to a 5 min heating process at 95 °C. Subsequently, equal quantities of protein were loaded and electrophoretically separated onto 12% sodium dodecyl sulfate-polyacrylamide (SDS) gels. Afterward, all proteins were transferred onto a polyvinylidene fluoride membrane. The membrane was then subjected to an overnight incubation at 4 °C with specific primary antibodies, namely SM22α (Abcam ab14106, 1:10,000), αSMA (Abcam ab124964, 1:1000), αTubulin (Abcam ab52866, 1:5000), and GAPDH (CST, 5174S, 1:5000). Following the primary antibody incubation, the membranes were exposed to the corresponding secondary antibodies. Protein bands were subsequently visualized using a chemiluminescent reagent (Thermo, 34580, Waltham, MA, USA) in conjunction with the Chem-iDoc™ Imaging System (12003153 BioRad, Hercules, CA, USA).

### 2.8. Real-Time Quantitative PCR (Q-PCR)

The aortic tissues (30 mg for each group) were subjected to homogenization employing an automated bead homogenizer. Following the manufacturer’s protocol, total RNA was extracted from the aortic tissue utilizing TRIzol (15596026 Thermo). The RNA concentration was determined as follows: A total of 2 µL of the sample was loaded following the NanoDrop (NDONEW, Thermo) instructions, and the measure button was clicked. Upon completion of the reading, the A260/A280 and A260/A230 ratios were recorded, along with the RNA recovery amount (in ng/µL). Subsequently, 1 μg of RNA samples underwent reverse transcription into cDNA through the utilization of the HiScript III RT SuperMix for qPCR kit (R323-01 Vazyme, Nanjing, China). The resultant cDNA was then utilized in real-time quantitative PCR with SYBR qPCR Master Mix (Q711-02 Vazyme) for the amplification of the target mRNA. The 2^−ΔΔCT^ relative quantification method was employed to estimate the amount of target mRNA in the samples. Each sample was amplified with at least three technical replicates. The primer sequences utilized for Q-PCR are shown in Table 1.

### 2.9. Statistical Analysis

The data presented in this study represent the mean values derived from a minimum of five biological replicates or biologically independent experiments. Statistical analyses were conducted using GraphPad Prism 8.0 (GraphPad Software 9.0, San Diego, CA, USA). Two-group comparisons were performed using unpaired 2-tailed t-test for data that passed the normality test. In experiments involving two factors, we conducted a two-way ANOVA, followed by a Bonferroni post hoc analysis. Survival curves were subjected to analysis utilizing the log-rank (Mantel–Cox) test. In all cases, statistical significance was defined as *p* < 0.05.

## 3. Results

### 3.1. Indole-3-aldehyde (3-IAId) Significantly Mitigates β-Aminopropionitrile Monofumarate (BAPN)-Induced Aortic Dissection (AD) Development in Mice

**Figure 1:**
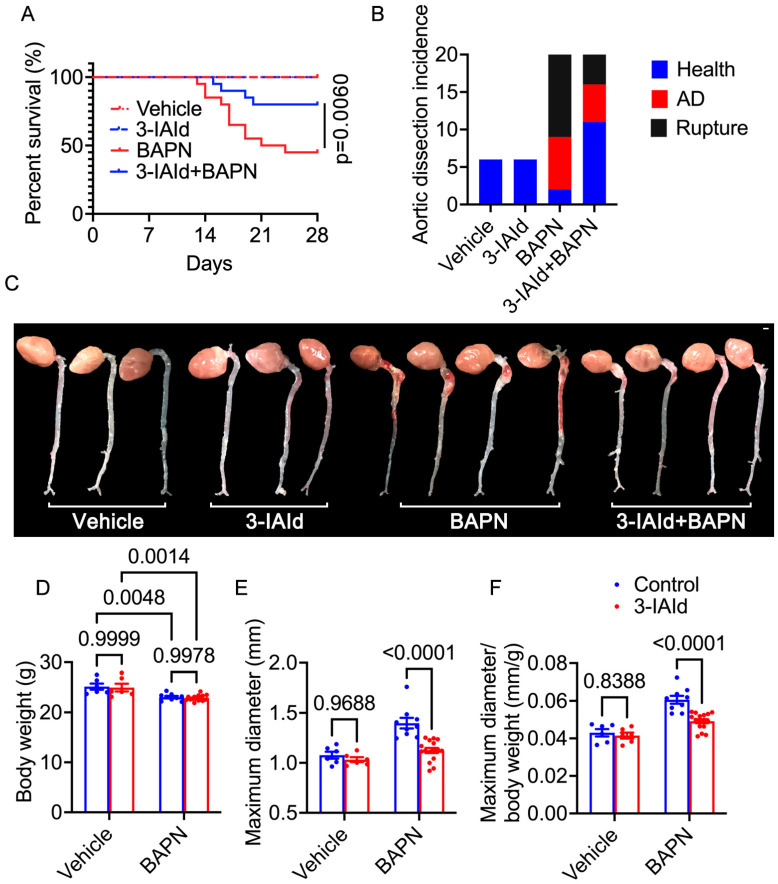
Aortic dissection incidence. (**A**) The survival rate was estimated by the Kaplan–Meier method and compared by log-rank test (*n* = 6 for vehicle and 3-IAId group, *n* = 20 for BAPN and 3-IAId + BAPN group). (**B**) AD incidence. (**C**) Representative macrographs of the aorta. (**D**), Body weight. (**E**), maximum diameter. (**F**), Maximum diameter normalized to body weight. Blue represents control group and red represents 3-IAId-treated group in histograms.

**Figure 2:**
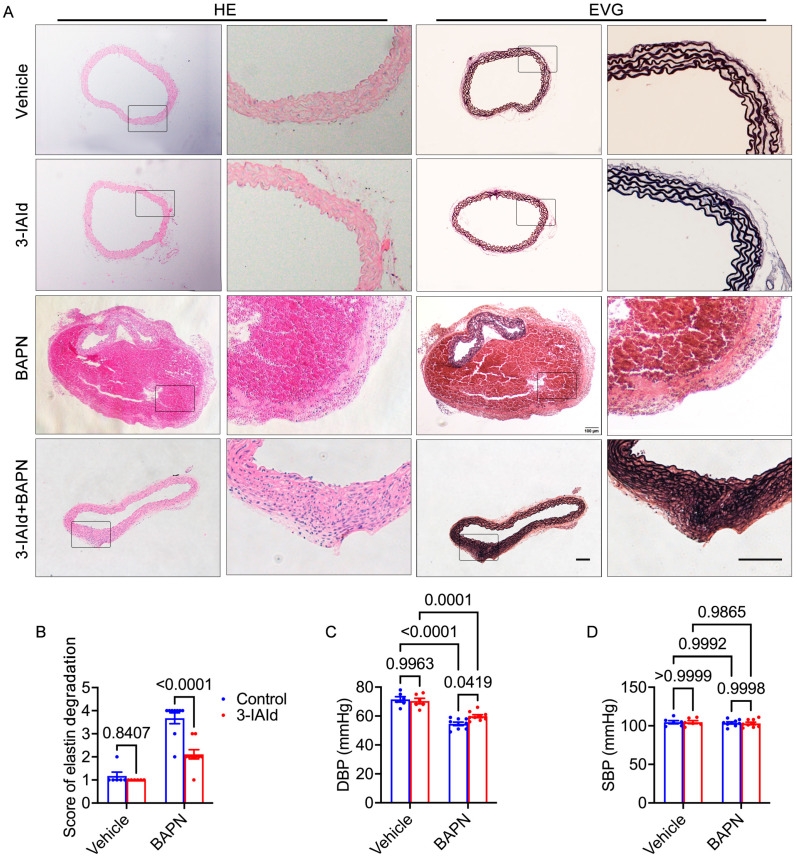
Pathological staining analysis after animal experiment end (BAPN-induced 28 days). (**A**) Representative images show hematoxylin and eosin (HE) and elastic Van Gieson (EVG) staining in paraffin sections from each group. Eosin is pink and stains proteins nonspecifically; nuclei are stained blue; elastic fibers are stained black. Scale bar, 100 μm, 100 μm. (**B**) Quantification of elastin degradation in paraffin sections: *n* = 6 for vehicle and 3-IAId group; *n* = 20 for BAPN and 3-IAId + BAPN group. (**C**,**D**) Systolic and diastolic blood pressure. Blue represents control group and red represents 3-IAId-treated group in histograms.

To investigate the impact of 3-IAId on AD, in vivo experiments were conducted using a BAPN-induced AD mouse model. The mice were divided into four groups: control group (*n* = 6), 3-IAId-treated group (*n* = 6), BAPN group (*n* = 20), and 3-IAId + BAPN group (*n* = 20). The survival curve revealed that 3-IAId treatment significantly improved the survival rate compared to the BAPN group (*p* = 0.0060, Figure 1A). Over the 28 days of BAPN administration, 55% (*n* = 11) of the BAPN group mice and 20% (*n* = 4) of the BAPN + 3-IAId group mice died from rupture (Figure 1A,B). Additionally, 35% (*n* = 7) of the BAPN group mice and 25% (*n* = 5) of the BAPN + 3-IAId group mice experienced AD without rupture (Figure 1C). Body weight measurements indicated that BAPN treatment significantly decreased body weight (Figure 1D). Maximal aortic diameter measurement and maximal aortic diameter normalized to body weight at day 28 after modeling demonstrated that BAPN treatment significantly increased the aortic diameter compared to the non-BAPN-treated control groups, while 3-IAId treatment mitigated BAPN-induced aortic dilation compared to BAPN-treated controls (Figure 1E,F). Hematoxylin and eosin along with elastic Van Gieson staining demonstrated that BAPN increased dissecting aneurysm formation and elastin disarray and degradation, which were alleviated in BAPN-treated 3-IAId mice (Figure 2A,B). Control and 3-IAId mice without BAPN treatment showed no difference in aortic wall thickness, diameter, and TAD occurrence (Figure 1A–C and Figure 2A). BAPN treatment reduced diastolic blood pressure (DBP) (Figure 2C) due to aortic sclerosis after TAD, whereas systolic blood pressure (SBP) remained relatively unchanged (Figure 2D). 3-IAId treatment rescued DBP in mice (Figure 2C). These findings indicate the beneficial effect of 3-IAId treatment in preventing AD formation.

### 3.2. 3-IAId Inhibits the Transition of Contractile VSMCs to Synthetic VSMCs

**Figure 3:**
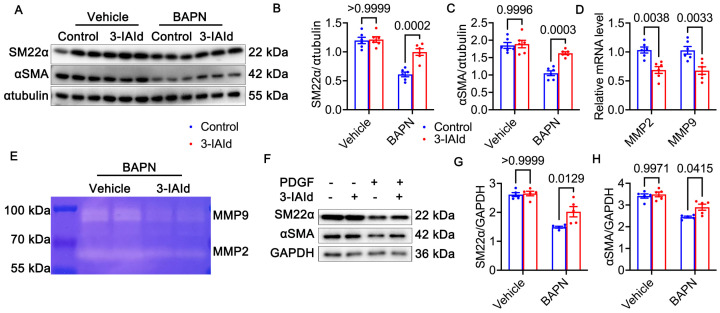
Phenotype switch of vascular smooth muscle cells (VSMCs) in vivo and in vitro. (**A**–**C**) The protein levels and quantitation of VSMC contractile markers (SM22α and α-SMA) in mice aorta (*n* = 6 per group). Two-way ANOVA with the Bonferroni correction. (**D**) mRNA levels of MMP2 and MMP9 in mice aorta (*n* = 6 per group). Two-way ANOVA with the Bonferroni correction. (**E**) Representative images of MMP-2 and MMP-9 in each group obtained by a gelatin zymogram. (**F**–**H**) The protein levels and quantitation of VSMC contractile markers (SM22α and α-SMA) in VSMCs of each group (*n* = 5 per group). Blue represents control group and red represents 3-IAId treated group in histograms.

Vascular smooth muscle cells (VSMCs) constitute the predominant cellular component within the medial layer of the aorta, thus imparting structural and functional integrity to the aortic wall. In response to environmental stimuli and mechanical stresses, VSMCs exhibit phenotypic plasticity, transitioning between a contractile state and a synthetic state, thereby influencing the pathogenesis of aortic diseases, including AD [25]. To assess alterations in the contractile phenotype of aortic tissues, we conducted Western blot analyses to detect the expression of contractile markers, specifically α-smooth muscle actin (α-SMA) and smooth muscle protein 22 alpha (SM22α). Remarkably, treatment with BAPN resulted in a substantial downregulation of both α-SMA and SM22α expression. Importantly, co-administration of 3-indoleacetic acid (3-IAId) effectively restored the diminished expression of α-SMA and SM22α (Figure 3A–C). Furthermore, we conducted gelatin zymography and quantitative polymerase chain reaction (qPCR) analyses to evaluate the levels of matrix metalloproteinase-2 (MMP-2) and matrix metalloproteinase-9 (MMP-9) within the aortic tissue. Intriguingly, the combined treatment with 3-IAId and BAPN resulted in a significant reduction in the expression of both MMP-2 and MMP-9 compared to the group treated solely with BAPN (Figure 3D,E). BAPN, a potent inhibitor of Lysyl-oxidase (LOX), increases the risk of both AD and AAA [26], accompanied by histological signs such as elastic fiber breaks, thrombus formation, and leukocyte infiltration [27]. LOX, an enzyme secreted by VSMCs, is responsible for collagen crosslinking between collagens. Loss-of-function mutation of LOX leads to TAA and dissection in humans [28]. The loss of structural organization in the aorta results in a weakened vessel and subsequent aortic dilation. Maintaining the contractile force of VSMCs is vital for both the function and structure of the aorta. Any disruption in contractile VSMC force generation could lead to the development of TAA and aortic dissection [29]. Platelet-derived growth factor (PDGF) is known to play a role in regulating the transition of VSMCs from a contractile phenotype to a synthetic one [30]. To validate these results above, in vitro experiments were performed using hVSMCs treated with PDGF to induce a phenotypic transformation. PDGF treatment resulted in a notable decrease in α-SMA and SM22α levels in SMCs, which were effectively restored by 3-IAId (Figure 3F–H). Overall, these results suggest that 3-IAId may play a role in preventing VSMC phenotype transformation signaling, thereby potentially mitigating aortic degeneration.

### 3.3. 3-IAId Blunts Extracellular Matrix Degradation of the Aorta

**Figure 4:**
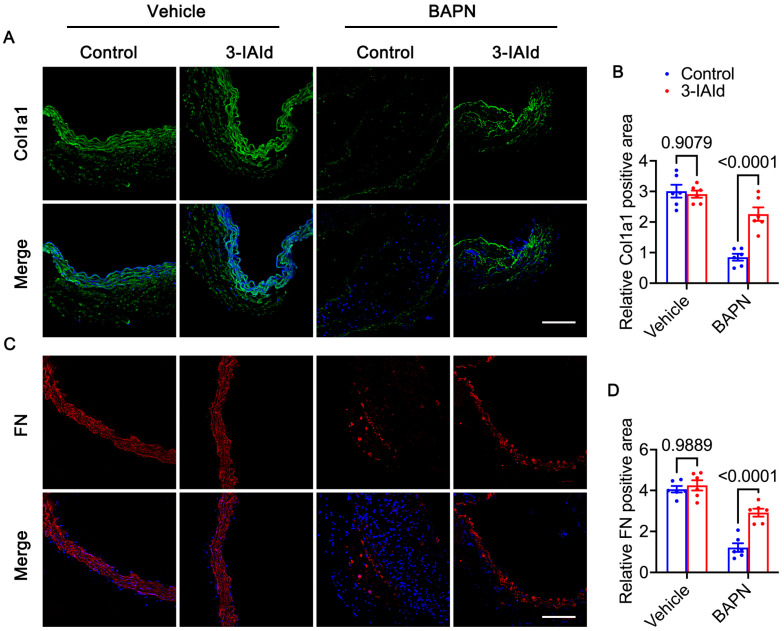
Extracellular matrix degradation of the aorta. (**A**,**B**) Immunofluorescence staining and quantification of Col1a1 (green) in aortas of each group. Nucleus were stained with DAPI (blue). Scale bars: 50 μm. (**C**,**D**) Immunofluorescence staining and quantification of Fibronectin (red) in aortas of each group. Nucleus were stained with DAPI (blue). Scale bars: 50 μm. *n* = 6 per group. Blue represents control group and red represents 3-IAId treated group in histograms.

The extracellular matrix (ECM) of the vascular wall primarily comprises elastic fibers and collagen fibers, which play a crucial role in both development and maintaining structural integrity [31]. The degradation of ECM can result in aortic dissection and the formation of aneurysms [21]. To gain deeper insights into the mechanism by which 3-IAId mitigates the risk of AD formation, an investigation was conducted to assess the deposition of collagen type I (COL1a1) and fibronectin (FN) within aortic lesions using immunofluorescence (IF) staining. The obtained results reveal a substantial increase in the density of COL1a1 and FN in the group treated with 3-IAId when compared to the control group, 28 days post-modeling. Notably, under physiological conditions, the levels of COL1a1 and FN remained comparable between the control and 3-IAId groups (Figure 4A–D). These findings suggest that the reduced incidence of ruptures observed with 3-IAId treatment can be attributed, in part, to the mitigation of ECM degradation.

### 3.4. 3-IAId Alleviates Inflammation in the Aorta

**Figure 5:**
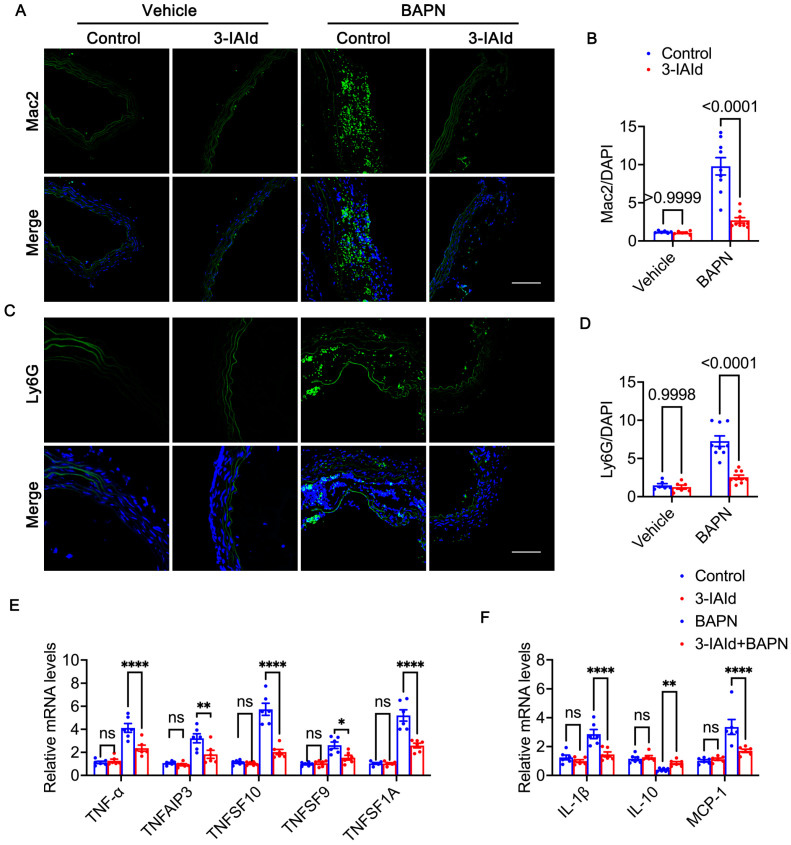
Vascular inflammation of the aorta. (**A**) Macrophage (stained in green) immunostaining of thoracic aortic sections in each experimental group. Nuclei were counterstained with DAPI (blue). Scale bars: 50 μm. (**B**) Quantification of Mac2-positive cells in each experimental group (*n* = 9). (**C**) Neutrophil (stained in green) immunostaining of thoracic aortic sections in each experimental group. Nuclei were counterstained with DAPI (blue). Scale bars: 50 μm. (**D**) Quantification of Ly6G-positive cells in each experimental group (*n* = 9). (**E**) Relative mRNA levels of *TNFSF10, TNFSF9, TNF-α*, *TNFAIP3*, and *TNFSF1A* in the mouse aorta (*n* = 6 per group), determined using a two-way ANOVA with Bonferroni multiple comparison test. (**F**) Relative mRNA levels of *IL-10, IL-1β* and *MCP-1* in the mouse aorta (*n* = 6 per group). Asterisks indicate *p*-values: * indicates a *p*-value less than 0.05, ** indicates a *p*-value less than 0.01, and **** indicates a *p*-value less than 0.001. ns indicates no significant difference.

In the context of AD, inflammation plays a pivotal role by perturbing the aortic wall’s homeostasis, culminating in VSMC apoptosis and ECM degradation [32]. The onset of AD is characterized by a substantial infiltration of macrophages into aortic wall lesions [33,34]. Our investigation has elucidated that treatment with BAPN resulted in an augmentation of the green fluorescence signal, indicative of macrophages (Mac-2) and neutrophil cells (Ly6G). Conversely, administration of 3-IAId notably curtailed the infiltration of both macrophages and neutrophils. This observation underscores the counteractive effect of 3-IAId against BAPN-induced inflammatory cell infiltration (Figure 5A–D). Numerous inflammatory factors, such as interleukin-6 (IL-6) and tumor necrosis factor-α (TNF-α), are released during the progression of AD [35]. Our qPCR analysis unveiled downregulation in the transcriptional levels of several tumor necrosis factors, including TNF-α, TNFAIP3, TNFSF10, TNFSF9, and TNFSF1A, within the aortic tissue of the BAPN + 3-IAId group compared to the BAPN group (Figure 5E). Furthermore, specific cytokines and chemokines play a pivotal role in facilitating the recruitment of inflammatory cells into the aortic media [33]. Our data demonstrated a reduction in interleukin and chemokine levels, notably IL-1β and MCP-1, within the aortic tissue of the BAPN + 3-IAId group in contrast to the BAPN group. Conversely, IL-10 exhibited an opposing trend (Figure 5F). In summary, treatment with 3-IAId effectively attenuated the extent of the inflammatory response by mitigating macrophage and neutrophil infiltration and suppressing the expression of cytokines and chemokines emanating from these inflammatory cells. Consequently, this therapeutic intervention exhibited a mitigating effect on the progression of aortic dissection (AD).

## 4. Discussion

Our research findings provide compelling evidence that treatment with 3-IAId can effectively alleviate the progression of aortic dissection (AD) in a BAPN-induced AD model. This study represents the first investigation into the role of 3-IAId in AD progression through nutritional intervention. The evidence presented, including improved survival rates, alleviated AD formation and rupture, reduced extracellular matrix (ECM) degradation, and downregulated VSMC phenotypic switch markers, strongly supports the successful intervention of 3-IAId in AD progression. Furthermore, 3-IAId has beneficial effects by downregulating aortic inflammation through the attenuation of macrophage and neutrophil infiltration, leading to a reduction in ECM degradation and VSMC phenotype transformation. Our study underscores the critical role of 3-IAId in AD progression and identifies a promising nutritional intervention strategy for AD prevention.

The essential amino acid, tryptophan, is exclusively obtained through dietary intake [36]. Tryptophan metabolism in various tissues is associated with diverse physiological functions [37]. One of the tryptophan metabolites produced by the gut microbiota, Indole-3-aldehyde (3-IAId), also known as Indole-3-carbaldehyde and 3-formylindole, is abundantly generated by the commensal bacterium *Lactobacillus reuteri* under conditions of unrestricted tryptophan availability. 3-IAId acts as a ligand of the aryl hydrocarbon receptor (AhR) [38]. Previous studies have demonstrated that another tryptophan metabolite, 3-IAld, ameliorates conditions such as EAE and colitis in an AhR-dependent manner [39,40].

Additionally, 3-IAld has been shown to reduce the expression of proinflammatory cytokines IL-6 by murine macrophages in response to stimulation by heat-killed M. tuberculosis sonicate (Mtb) [17]. In dermatitis, 3-IAId significantly attenuates skin inflammation induced by MC903 in mice with AD-like symptoms. This effect is blocked by an AHR antagonist and abolished in AHR-null mice [41]. Studies also suggest that targeted delivery of 3-IAld through pharmaceutics may have therapeutic benefits in murine models of metabolic and organ inflammatory pathology. Notably, a pantissue AHR signature has highlighted 3-IAld as one of the metabolites downstream of L-amino acid oxidase catabolism of tryptophan, associated with AHR-driven cancer cell motility and immunosuppression [42]. Corresponding with these prior findings, our study provides further evidence of the protective role of 3-IAId in aortic dissection.

The development of aortic dissection involves a two-step process: firstly, the occurrence of aortic intima tear or ulceration results in the rupture of the aortic intima, allowing for the entry of blood into the tunica media; secondly, the rupture of nourishing vessels within the aortic media leads to the leakage of blood into the media. Subsequent to the formation of dissection, aortic inflammation triggers dilation, subsequently leading to the rupture of the aorta [43]. The adventitia of the aortic wall experiences infiltration by inflammatory cells, which in turn produce enzymes, cytokines, and chemokines, thereby causing degradation of the aortic extracellular matrix (ECM) and direct interaction with vascular smooth muscle cells (VSMCs) [44,45,46,47]. Ultimately, the inflammatory response induces vascular remodeling and progressive degeneration of the aortic wall, potentially culminating in aortic dissection and rupture [48]. In our study, it was observed that treatment with 3-IAId resulted in a significant reduction in inflammatory cell infiltration, a downregulation in the expression of inflammatory markers, and a mitigation of the progression of aortic dissection. These findings strongly indicate that 3-IAId effectively prevents the development of aortic dissection by attenuating the inflammatory response within the aortic wall.

Vascular smooth muscle cells (VSMCs) play a pivotal role in the maintenance of vascular homeostasis and contractility [49]. During the progression of aortic dissection, VSMCs undergo a phenotypic switch from a contractile to a synthetic phenotype [50,51]. This phenotypic transition leads to ECM degradation, promoting detachment of VSMCs from ECMs and accelerating their migration and apoptosis [49]. Our study unequivocally demonstrates that treatment with 3-IAId leads to a substantial reduction in the expression of synthetic markers within VSMCs, concomitantly with an upregulation in the expression of contractile markers. This effect effectively mitigates the vascular dysfunction induced by BAPN administration. These findings provide compelling evidence that the 3-IAId-mediated reduction of tissue inflammation plays a pivotal role in partially ameliorating the progression of AD by inhibiting the synthetic phenotype of VSMCs. However, it is worth noting that the precise molecular mechanisms through which 3-IAId exerts its inhibitory effects on the phenotypic transition of VSMCs necessitate further in-depth investigation.

In addition to their pivotal role in maintaining vascular homeostasis, the extracellular matrix (ECM) assumes a critical function in preserving the structural integrity and functionality of the arterial wall, primarily by endowing it with elasticity and distensibility. Furthermore, the ECM serves as a reservoir for signaling molecules and actively engages with adhesion molecules to relay indispensable cellular signals [52]. Throughout the progression of abdominal aortic aneurysm (AD), there is an upregulation in the degradation of the ECM and the expression of ECM enzymes, notably including MMP-9, MMP-2, and ADAMTs [46,53,54]. In our investigation, treatment with 3-IAId resulted in the downregulation of ECM enzymes, such as MMP-2 and MMP-9, and attenuated the characteristic manifestations of ECM degradation. These manifestations encompassed a reduction in elastin fiber fragmentation, collagen deposition, and proteoglycan accumulation. In line with prior research, our findings underscore the efficacy of 3-IAId intervention in inhibiting ECM degradation within the BAPN-induced AD model. However, it is imperative to note that further investigations are imperative to gain a comprehensive understanding of the underlying mechanisms governing this process.

While our study has elucidated the role of 3-IAId in AD progression through in vivo experiments, it remains uncertain whether 3-IAId plays the same role in in vitro cell experiments. Notably, conducting in vitro experiments presents challenges due to the complex regulation of 3-IAId in AD, which involves immune cells, inflammation-related factors, and VSMCs. Furthermore, we acknowledge that using male mice in our experiments was to reduce hormonal variability, and the results may not directly apply to female mice. Nevertheless, our results strongly indicate the crucial role of 3-IAId in improving AD outcomes.

In conclusion, our study provides compelling evidence for the protective effects of 3-IAId in AD development. Treatment with 3-IAId effectively reduces the infiltration of macrophages, downregulates aortic inflammation, and inhibits the phenotypic switch of VSMCs and ECM degradation, leading to the attenuation of AD progression. Overall, our findings suggest that 3-IAId holds promise as an effective and promising strategy for preventing and treating aortic dissection.

## Figures and Tables

**Table 1:** Primers for real-time PCR analysis in mice.

Gene	Forward (5′-3′)	Reverse (5′-3′)
IL-1β	GCAACTGTTCCTGAACTCAACT	ATCTTTTGGGGTCCGTCAACT
IL-10	GCTCTTACTGACTGGCATGAG	CGCAGCTCTAGGAGCATGTG
MCP-1	TTAAAAACCTGGATCGGAACCAA	GCATTAGCTTCAGATTTACGGGT
TNF-α	CCCTCACACTCAGATCATCTTCT	GCTACGACGTGGGCTACAG
TNFSF10	ATGGTGATTTGCATAGTGCTCC	GCAAGCAGGGTCTGTTCAAGA
TNFSF9	CGGCGCTCCTCAGAGATAC	ATCCCGAACATTAACCGCAGG
TNFRSF1A	CCGGGAGAAGAGGGATAGCTT	TCGGACAGTCACTCACCAAGT
TNFAIP3	GAACAGCGATCAGGCCAGG	GGACAGTTGGGTGTCTCACATT
MMP2	CAAGTTCCCCGGCGATGTC	TTCTGGTCAAGGTCACCTGTC
MMP9	CTGGACAGCCAGACACTAAAG	CTCGCGGCAAGTCTTCAGAG

## Data Availability

All data have been presented in the text. Data sharing is not applicable to this article.
